# Geospatial risk prediction of hookworm infection and intensity among school-aged children in Dak Lak province, Vietnam

**DOI:** 10.1371/journal.pntd.0014079

**Published:** 2026-03-12

**Authors:** Tin N. D. Pham, Adam W. Bartlett, Katrina Blazek, Sze Fui Hii, Vito Colella, Dinh Ng-Nguyen, Susana Vaz Nery

**Affiliations:** 1 School of Population Health, University of New South Wales, Sydney, Australia; 2 Global Health Program, Kirby Institute, University of New South Wales, Sydney, Australia; 3 Melbourne Veterinary School, University of Melbourne, Melbourne, Australia; 4 Faculty of Animal Sciences and Veterinary Medicine, Tây Nguyên University, Buôn Ma Thuột, Vietnam; University of Passo Fundo: Universidade de Passo Fundo, BRAZIL

## Abstract

**Background:**

Hookworms remain problematic in Dak Lak province, Vietnam, despite a school preventive chemotherapy (PC) program since 2007. As hookworms depend on favourable ecological conditions, geospatial modelling incorporating environmental and climatic variables can predict high-risk areas for targeted interventions. This study provides geospatial risk predictions for hookworm infection and intensity among school-aged children in Dak Lak.

**Methods:**

Hookworm infection status and intensity from 7,964 school-aged children from 64 schools collected in 2019–2020 during the Community Deworming for STH trial was combined with environmental and climatic data to develop risk prediction models for (i) overall hookworm infection and (ii) moderate-and-heavy intensity (MHI) *Necator americanus* infection. Environmental and climatic predictors for the multivariable generalised linear models were selected by identifying the model with the lowest Akaike Information Criterion. Semivariograms were examined for residual spatial autocorrelation, and if present, was accounted for using Matérn’s covariance. Regression coefficients were used to predict overall hookworm and MHI *N. americanus* infection risk across Dak Lak province pre- and post-intervention.

**Results:**

Temperature, precipitation, soil and vegetation variables were included in the hookworm model, while temperature and precipitation variables were included in the MHI *N. americanus* model. Most of Dak Lak had a predicted hookworm risk of 10–15% pre- and post-intervention, with high-risk hotspots in southern and eastern parts. Moderate-and-heavy intensity *N. americanus* infection risk pre-intervention was higher than 2% throughout the province and considerably reduced to small pockets in southeastern, central and northern areas post-intervention.

**Conclusion:**

School-based PC should be delivered across Dak Lak at least annually, in keeping with World Health Organization recommendations. However, several hotspots have been identified that would benefit from increased frequency of school-based PC or community-wide mass drug administration.

## Author summary

This geospatial analysis incorporated georeferenced parasitological data from 7,964 school-aged children across 64 schools involved in a large clinical trial with open-source environmental and climatic data to develop spatial risk prediction models for overall hookworm infection and moderate-and-heavy intensity hookworm infection pre- and post-intervention (community-wide or school-based deworming) across Dak Lak province, Vietnam. Our risk prediction maps showed most of Dak Lak had a predicted hookworm risk of 10–15% pre- and post-intervention, with high-risk (>50% prevalence) hotspots in southern and eastern parts of the province. There were considerable portions of the province with a moderate-and-heavy intensity *Necator americanus* infection risk higher than 2% pre-intervention, which considerably reduced post-intervention. These maps inform the implementation of geographically targeted control strategies, particularly for high-risk hotspots that would benefit from community-wide deworming to optimise reduction in hookworm infection and burden.

## Introduction

Soil-transmitted helminth (STH) infections are a pervasive public health problem especially in socioeconomically disadvantaged regions [1,2]. The major STH species include *Ascaris lumbricoides*, *Trichuris trichiura, Strongyloides stercoralis,* and hookworms, which comprise the species *Necator americanus, Ancylostoma duodenale* and *Ancylostoma ceylanicum.* These parasites can cause considerable morbidity, particularly in pregnant women and school-aged children including anaemia and malnutrition leading to poor growth and cognitive development [3–5]. The World Health Organization (WHO) has set targets for STH elimination as a public health problem, defined as <2% prevalence of MHI infections, by 2030 [6]. To achieve this, the WHO have provided guidance on preventive chemotherapy (PC) for school-aged children as the primary control strategy, with the frequency of PC based on STH prevalence [6]. Vietnam is a tropical country endemic for STHs, including in Dak Lak province, a socioeconomically disadvantaged region with poor water, sanitation and hygiene (WASH) infrastructure [7]. Pooled analyses on STH prevalence from studies published between 1990 and 2015 in Vietnam estimated hookworm prevalence of 29%, *A. lumbricoides* prevalence of 36% and *T. trichiura* prevalence of 52% [8]. As a result, considerable efforts have been allocated to STH control strategies, such as school-based PC and promotion of WASH practices in keeping with recommendations from the WHO [9]. In Dak Lak province, PC for school-aged children has been conducted twice a year from 2007, and once a year since 2019 either with albendazole or mebendazole [10,11]. With these decade-long efforts, the prevalence of *A. lumbricoides* and *T. trichiura* among school-aged children has dropped to very low levels in the province [12,13]. However, hookworm infections have remained a public health challenge, with persisting high prevalence and morbidity, as indicated by high rates of MHI infection [12,13].

In this context, the CoDe-STH trial was a cluster randomised controlled trial conducted from October 2019 to November 2020 in Dak Lak province, Vietnam, aimed to compare the effectiveness of community-wide mass drug administration (MDA) versus school-based PC in reducing overall hookworm prevalence and infection intensity (as a marker of morbidity) in school-aged children (age 6–11 years) [11,12]. This trial found no difference in impact between community-wide MDA and school-based PC in reducing overall hookworm prevalence, which may be in part due to the lower than expected pre-intervention prevalence, but found community-wide MDA to be more effective in lowering *N. americanus* infection intensity in schoolchildren compared to school-based PC [12]. This was supported by a cost-effectiveness analysis that found community-wide MDA to be more cost-effective than school-based PC in controlling STH burden [14].

Soil-transmitted helminths spend integral aspects of their life cycle in the environment, as such their transmission is influenced by environmental factors that range from climate-related variables, such as temperature and rainfall, to soil characteristics and vegetation [15]. Acknowledging these associations between ecological conditions and risk of STH infection, geospatial modelling presents as a promising approach to provide risk predictions for STH infections to inform implementation of control strategies [16–20]. This application of geospatial modelling has gained increasing recognition with the WHO recently incorporating such methods into recommendations for STH epidemiological surveys and impact assessments [6]. However, strengthening in-country capacity for geospatial modelling is required for many low- and middle-income countries that are endemic for STHs. In this context of persisting high prevalence and morbidity of hookworm, despite the long running school PC program, and the benefits shown for community-wide MDA to reduce *N. americanus* infection intensity, this geospatial analysis combined parasitological data collected from the CoDe-STH trial [12] with open-source environmental and climatic data to predict the risk of hookworm infection and MHI *N. americanus* infection to identify priority areas for targeted interventions.

## Methods

### Study location

**Fig 1:**
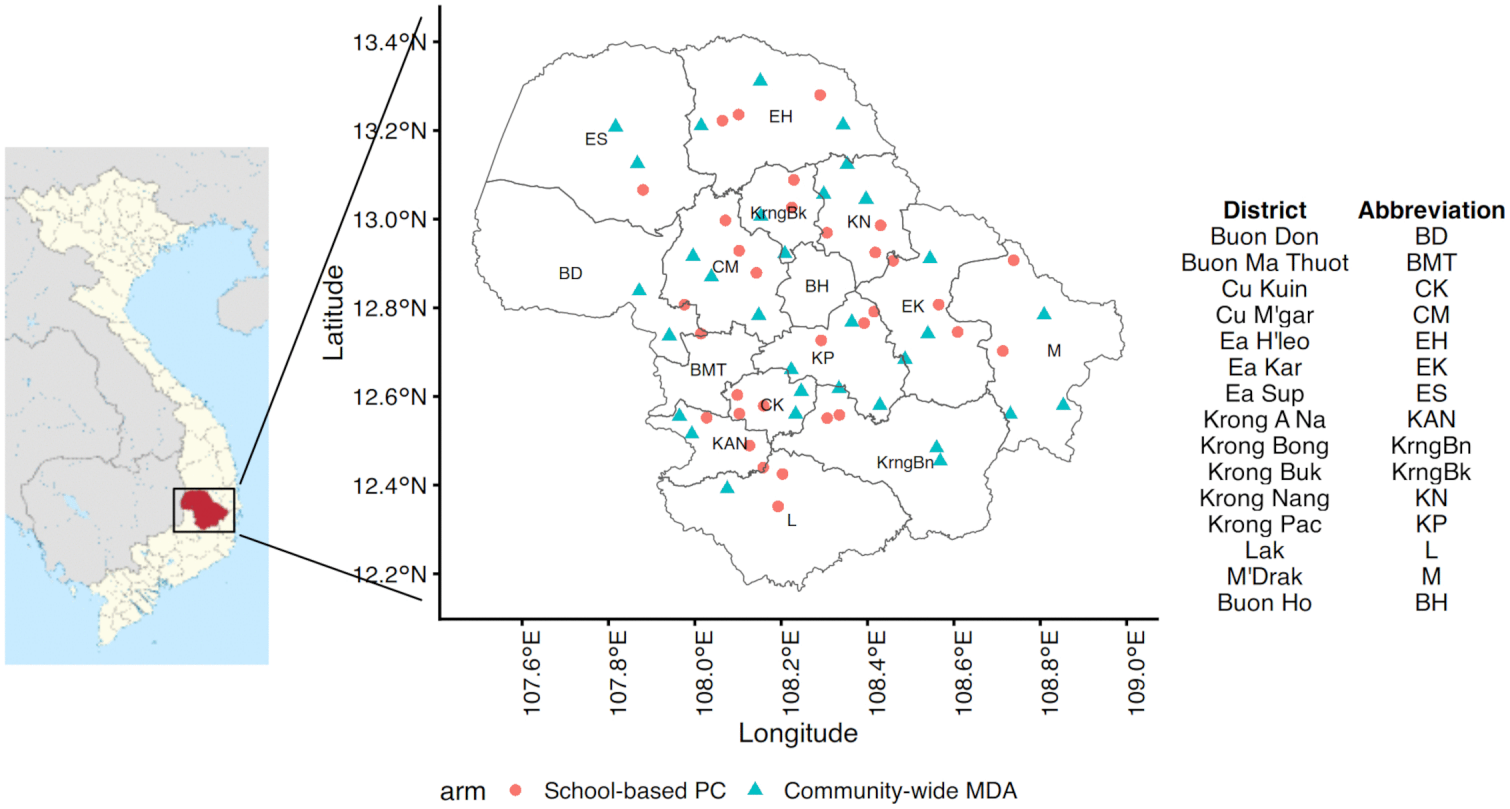
Location of Dak Lak province in Vietnam, and of study sites in relation to the province’s administrative divisions.

Dak Lak is a mountainous province located in the Central Highlands in Vietnam with a median elevation of 453 metres above sea level (Fig 1). The province exhibit patterns typical to tropical savanna climate zones – hot temperature year-round and ample precipitation concentrated in the rainy season – except for higher altitudes which have more subtropical characteristics according to Köppen’s climate classification.

Map of Vietnam adapted from Clarke *et al* [11]. Base-layer map provided by the Database of Global Administrative Areas (GADM): https://gadm.org/download_country.html; license: https://gadm.org/license.html.

### Data sources

Full details of the CoDe-STH trial are reported elsewhere [11]. Infection status pre-intervention and 12-months post-intervention for 5,093 children from 32 primary schools in the community-wide MDA arm and 4,955 children from 32 primary in the school-based PC arm were determined using quantitative polymerase chain reaction (qPCR) analysis of faecal samples, and was available for *A. lumbricoides, T. trichiura, S. stercoralis, A. duodenale, A. ceylanicum,* and *N. americanus*. Hookworm infection was defined as having either *A. duodenale, A. ceylanicum,* or *N. americanus* detected by qPCR [11]. Infection intensity was determined by converting qPCR cycle threshold (Ct) values to eggs per gram (EPG) using previously published conversion formulae [12]. These conversion formulae are derived from laboratory-based faecal seeding experiments with known quantities of species-specific STH eggs across light, moderate and heavy infection intensity according to WHO criteria [21]. In field studies, qPCR has generally been shown to be more sensitive than microscopy in detecting hookworm, and with greater infection intensity, including in Dak Lak province where qPCR was also found to have moderate to good agreement between qPCR and microscopy (Kato Katz and Sodium Nitrate Flotation) [22]. A main limitation of qPCR is the potential for embryonation in faecal specimens during collection and storage in the field and transportation to a central laboratory, leading to increased DNA content and overestimation of infection intensity. This is monitored by quality assurance through microscopy assessing for embryonation in a random sample of stool specimens and the application of adjusted conversion formulae to account for embryonation should this be detected. Embryonation had been detected in stool specimens collected for the CoDe-STH trial, as such embryonation-adjusted conversion formulae were used for this analysis as was done for the CoDe-STH trial [12]. Infection intensity estimates were only available for *A. lumbricoides*, *T. trichiura* and *N. americanus*, for which Ct to EPG conversion formulae have been established [21]. For hookworm, the focus of this analysis, two outcomes were investigated: (i) overall hookworm infection; and (ii) MHI *N. americanus* infection. In keeping with WHO recommendations, infection intensity was categorised into low intensity and MHI, based on the EPG thresholds [9]. For hookworms, light intensity infections range from 1 to 1,999 EPG, moderate intensity infections from 2,000–3,999 EPG, and heavy intensity infections from ≥ 4,000 EPG [9].

Extraction for bioclimatic, soil and elevation data were performed using the geodata package in R [23]. Bioclimatic variables were obtained from WorldClim database [24]. Soil characteristics were obtained from the International Soil Reference and Information Centre (ISRIC) SoilGrids database [25]. Elevation data were retrieved from the Shuttle Radar Topography Mission satellite dataset [26]. Vegetation data were obtained from Moderate Resolution Imaging Spectroradiometer (MODIS) satellite database as Normalised Difference Vegetation Index (NDVI) and Enhanced Vegetation Index (EVI) [27]. Data was collected from 1st June 2020 with a time resolution of 16 days and spatial resolution of 1km (30 arc-seconds).

### Statistical analysis

**Fig 2:**
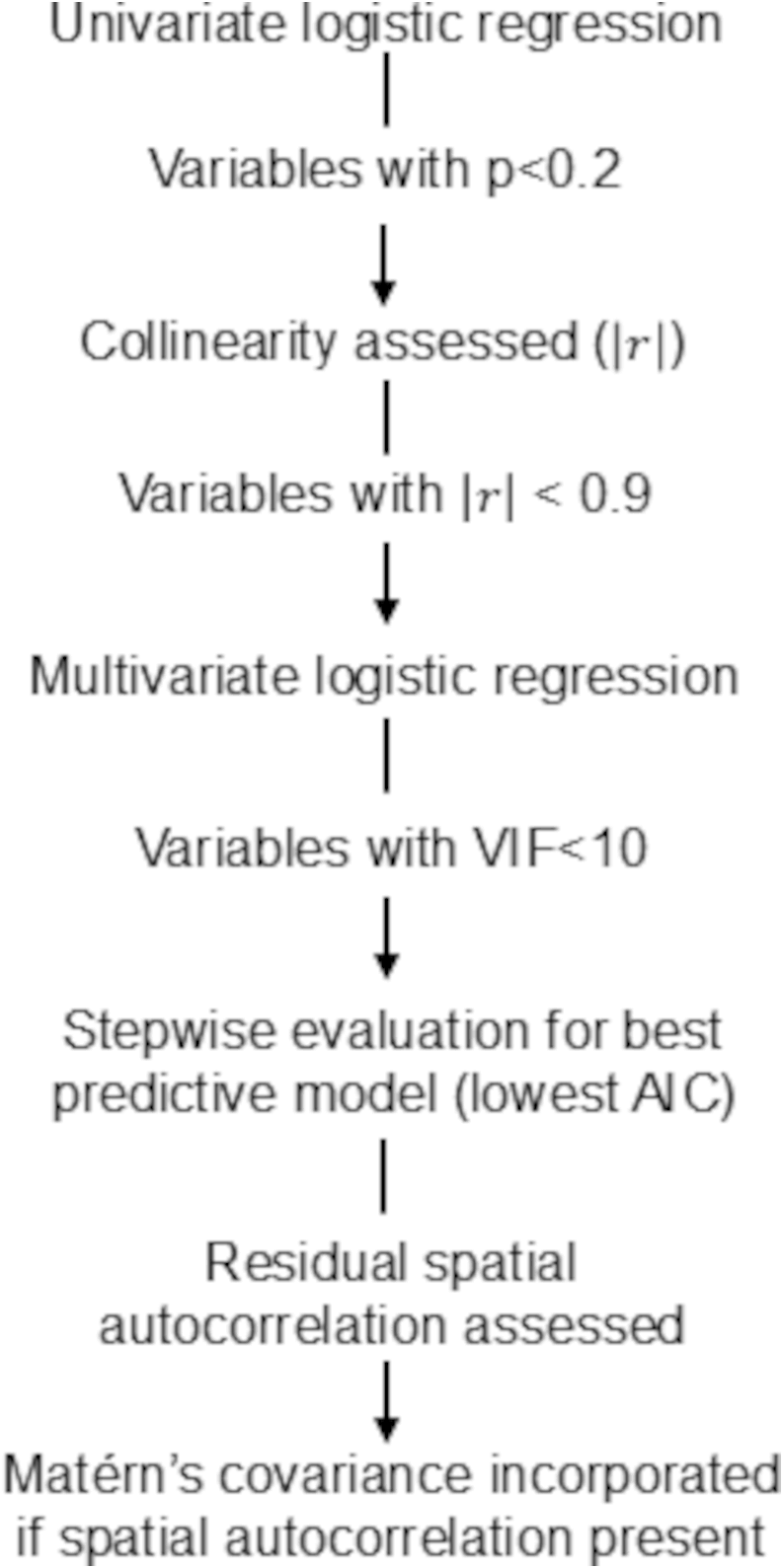
Flow diagram of modelling approach. AIC = Akaike Information. r = Pearson correlation coefficient. VIF = variance influence factor.

Statistical analyses were conducted in R statistical software, version 4.3.1. Descriptive statistics were used, adjusted for the school-level cluster design of the parasitological study. Prevalence was aggregated at the school level, and related statistics, including confidence intervals (CIs), were calculated accordingly. Univariable logistic regression analyses were performed for each environmental or climatic variable using pre-intervention hookworm data. Variables with a p-value < 0.2 were checked for collinearity, and for highly correlated variables(|r| > 0.9), the variable with the lower Akaike information criterion (AIC) was selected. These variables were then included in a multivariable generalised linear model (binomial family) and all variables with a variance inflation factor (VIF) <10 maintained. Backward-and-forward stepwise regression was then performed to obtain a model with the lowest AIC. Residual spatial autocorrelation was then evaluated using semivariograms, and if present, Matérn’s covariance was incorporated in the model to account for spatial effects [28] (Fig 2). Coefficients obtained from the final models were then used to develop risk predictions maps for overall hookworm prevalence and MHI *N. americanus* infection pre-intervention and 12-months post-intervention.

Residuals from each of the models were checked for deviation from normality by visual inspection of the Q-Q plot as well as the dispersion test and KS test. Model performance was measured using K-fold cross-validation where a subset containing 80% of the observations was used as training data, and the remaining observations as validation data. The root-mean-square error (RMSE) between the predicted and the observed data in the validation dataset was calculated. This process was repeated 10 times and the mean RMSE and standard deviation (SD) was calculated among the iterations.

### Ethics

Ethics approval for the parasitological data was granted by the University of New South Wales, Australia (HC190136) and Tay Nguyen University, Vietnam (1804/QĐ-ĐHTN-TCCB). Environmental and climatic data were open source. Verbal consent was obtained from either school headmasters of participating schools or hamlet leaders in the community to visit and approach families for written informed consent. Written informed consent was then obtained by parents or guardians of eligible children prior to undertaking data and stool sample collection [12].

## Results

### Descriptive statistics

**Fig 3:**
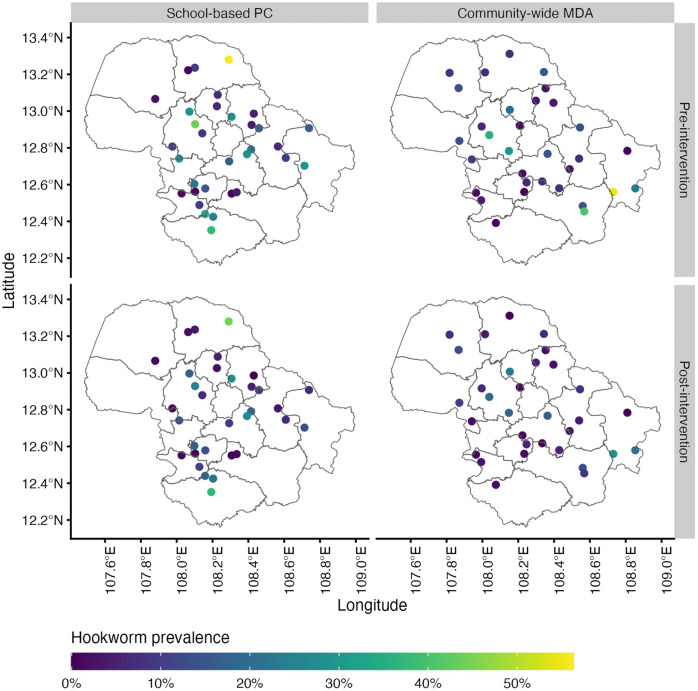
Hookworm prevalence measured by quantitative polymerase chain reaction at sampled schools, stratified by intervention (community-wide MDA or school-based PC) and timepoint (pre- or post-intervention). MDA = mass drug administration. PC = preventive chemotherapy. Base-layer map provided by the Database of Global Administrative Areas (GADM): https://gadm.org/download_country.html; license: https://gadm.org/license.html.

A total of 7,694 samples across 64 schools pre- and post-intervention were included in the analysis. The mean school cluster size was 120 participants (range 70, 135). The cluster-adjusted hookworm prevalence was 14.2% (school range 0%, 56.3%) pre-intervention and 8.6% (school range 0%, 44.4%) post-intervention. For MHI *N. americanus* infection, the cluster-adjusted prevalence was 3.3% (school range 0%, 17.3%) pre-intervention and 1.3% (school range 0%, 15.2%) at post-intervention. Overall hookworm prevalence and MHI *N. americanus* prevalence at each study site are presented in S1 Table, and illustrated in Fig 2 and Fig 3, respectively. Study sites were relatively evenly dispersed throughout the province, except for remote areas in the northwest and south-southeast corners where samples were not collected. Environment and bioclimatic features in Dak Lak across the province and at sampled schools are summarised in S2 Table.

### Geostatistical models

Variables included in the initial and final multivariable logistic regression models for overall hookworm prevalence and MHI *N. americanus* infection pre-intervention are shown in Table 1 and Table 2, respectively. For overall hookworm infection, a range of temperature, precipitation, soil and vegetation variables were included in the final model. For MHI *N. americanus* infection, both temperature and precipitation variables were included in the final model. Semivariograms from the residuals for each model showed autocorrelation (S1 Fig), as such Matérn’s covariance was incorporated into the models to account for spatial effects. Results from the respective univariable logistic regression analyses are presented in S3 Table.

**Table 1:** Non-spatial multivariable model for overall hookworm infection, using pre-intervention parasitological data.

	Initial model		Final model	
Variable	P-value	OR (95% CI)	P-value	OR (95% CI)
Mean Diurnal Range (°C)	<0.001	0.18 (0.13, 0‧26)	<0.001	0.20 (0.15, 0.28)
Isothermality (%)	0.053	1.09 (1.00, 1‧18)	0.091	1.07 (0.99, 1.16)
Max Temp. of Warmest Month (°C)	<0.001	0.67 (0.6, 0‧75)	<0.001	0.68 (0.61, 0.76)
Precipitation of Wettest Quarter (cm)	0.025	0.99 (0.98, 1.00)	0.051	0.99 (0.99, 1.00)
Precipitation of Driest Quarter (cm)	0.22	1.02 (0.99, 1.05)	–	–
Precipitation of Coldest Quarter (cm)	<0.001	1.07 (1.04, 1.09)	<0.001	1.06 (1.04, 1.08)
Soil pH	<0.001	0.06 (0.02, 0.15)	<0.001	0.05 (0.02, 0.11)
Soil organic carbon (dg/kg)	0.003	0.96 (0.93, 0.99)	0.002	0.96 (0.93, 0.98)
EVI (per 0.01)	<0.001	0.94 (0.93, 0.95)	<0.001	0.94 (0.93, 0.95)
AIC	1135.3		1134.7	

AIC = Akaike information criterion. CI = confidence interval. EVI = enhanced vegetation index. OR = odds ratio.

**Table 2:** Non-spatial multivariable model for moderate-and-heavy intensity *Necator americanus* infection, using pre-intervention parasitological data.

	Initial model		Final model	
Variable	P-value	OR (95% CI)	P-value	OR (95% CI)
Mean Diurnal Range (°C)	0.04	0.59 (0.36, 0.98)	0.04	0.60 (0.36, 0.98)
Temperature Seasonality (°C × 100)	0.18	0.99 (0.97, 1.01)	0.14	0.99 (0.97, 1.01)
Max Temp. of Warmest Month (°C)	<0.001	0.67 (0.55, 0.82)	<0.001	0.71 (0.61, 0.82)
Isothermality (%)	0.61	0.95 (0.80, 1.14)	–	–
Precipitation Seasonality (cm)	0.48	1.10 (0.84, 1.46)	–	–
Precipitation of Coldest Quarter (cm)	<0.001	1.08 (1.04, 1.12)	<0.001	1.08 (1.04, 1.12)
Soil organic carbon (dg/kg)	0.43	0.98 (0.94, 1.03)	–	–
AIC	497.4		492.7	

AIC = Akaike information criterion. CI = confidence interval. OR = odds ratio.

**Fig 4:**
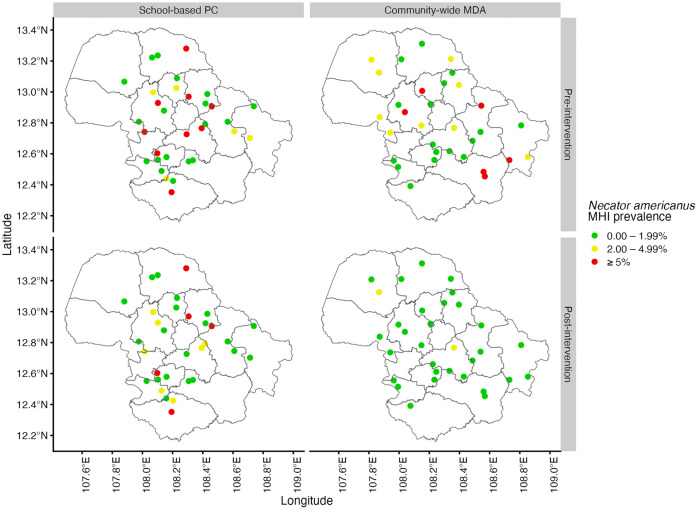
Moderate-and-heavy intensity *Necator americanus* infection prevalence at sampled schools, stratified by intervention (community-wide MDA or school-based PC) and timepoint (pre- or post-intervention). MDA = mass drug administration. MHI = moderate-and-heavy intensity. PC = preventive chemotherapy. Base-layer map provided by the Database of Global Administrative Areas (GADM): https://gadm.org/download_country.html; license: https://gadm.org/license.html.

**Fig 5:**
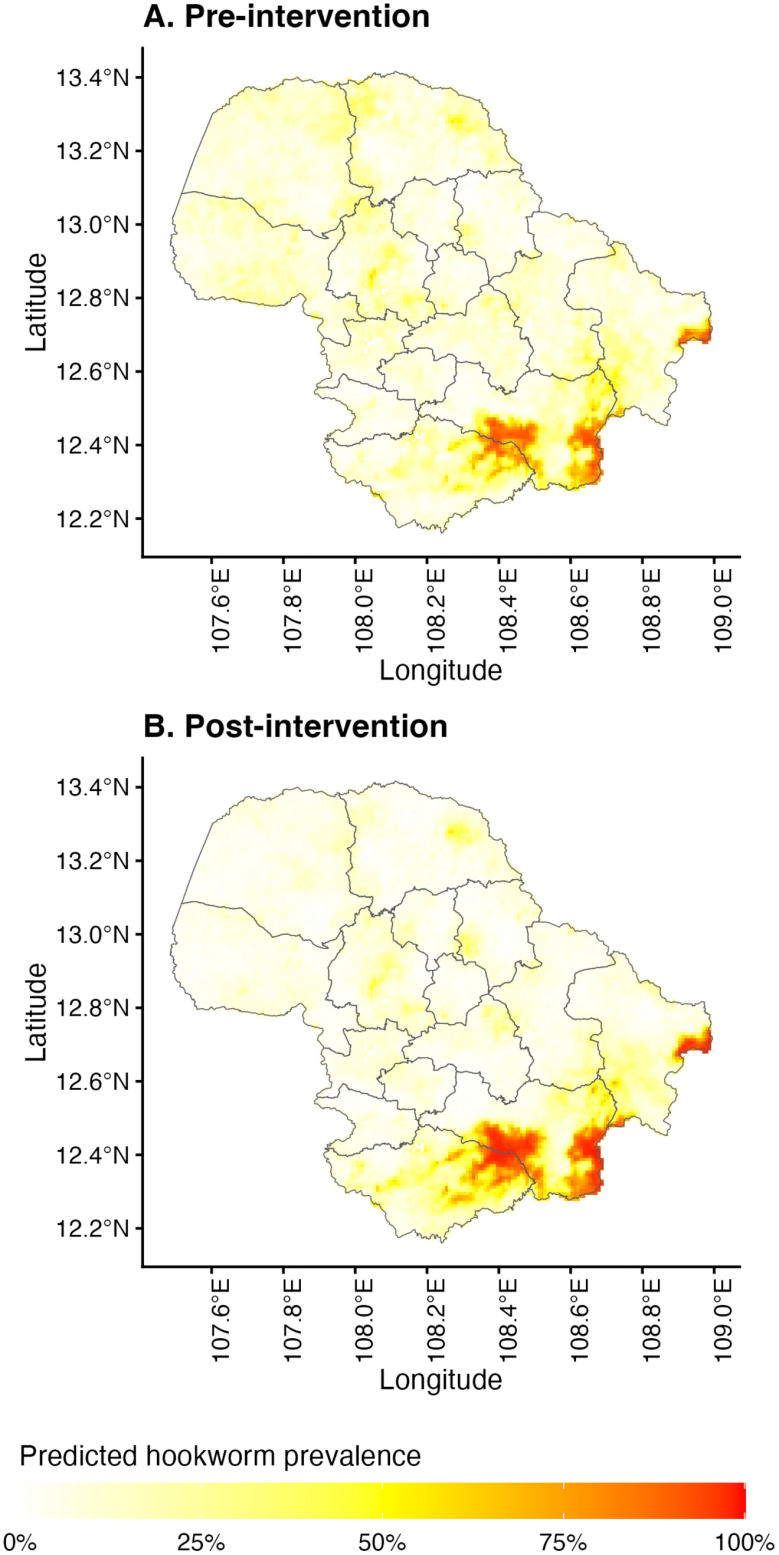
Predicted hookworm infection risk (A) pre- and (B) post-intervention. Base-layer map provided by the Database of Global Administrative Areas (GADM): https://gadm.org/download_country.html; license: https://gadm.org/license.html.

**Fig 6:**
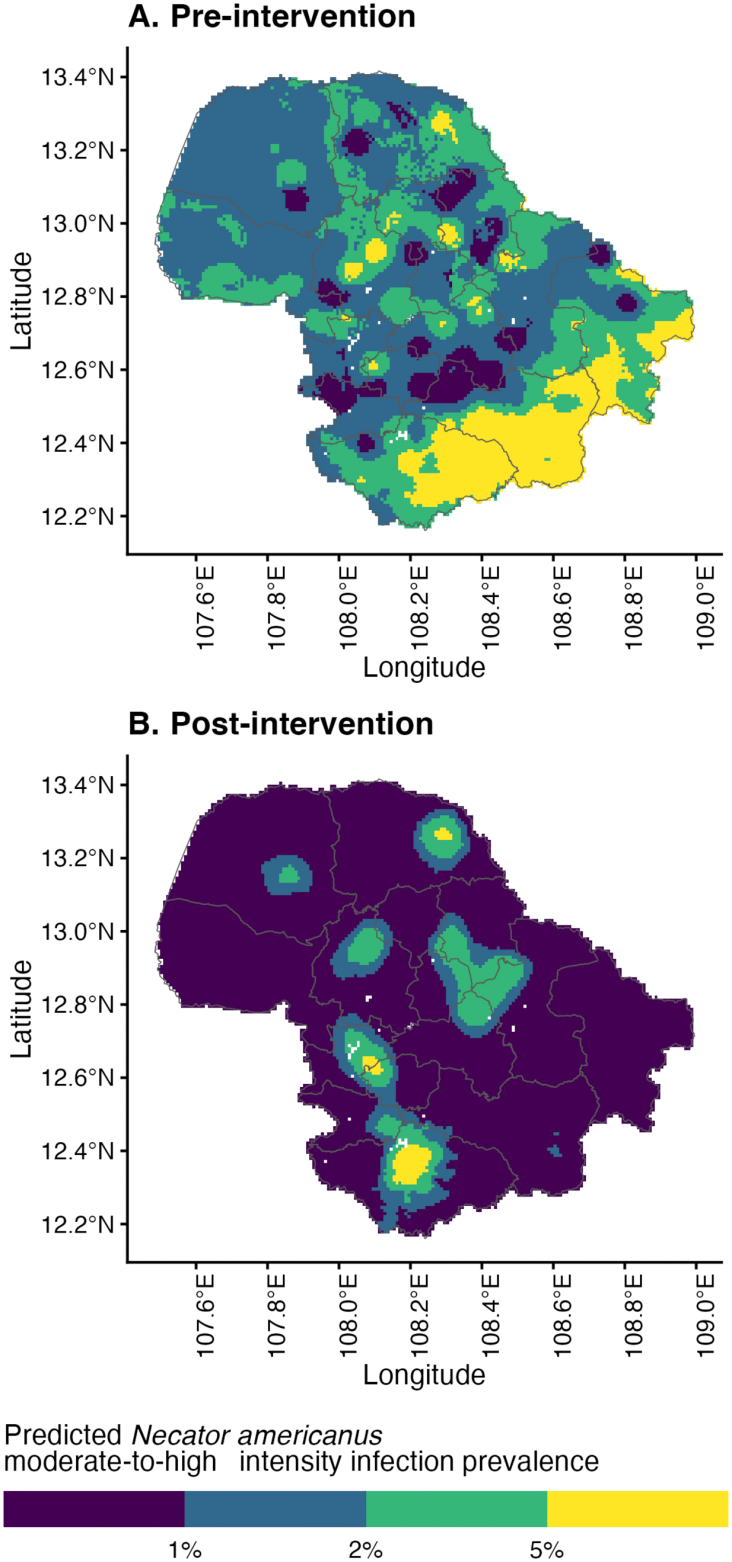
Predicted moderate-and-heavy intensity *Necator americanus* infection risk (A) pre- and (B) post-intervention. Base-layer map provided by the Database of Global Administrative Areas (GADM): https://gadm.org/download_country.html; license: https://gadm.org/license.html.

Using the regression coefficients, the predicted risk for overall hookworm infection and MHI *N. americanus* infection was mapped across Dak Lak province pre- and post-intervention (Fig 4). These predictive risk maps indicate a similar spatial distribution of overall hookworm prevalence pre- and post-intervention. Pre-intervention, southern areas were considered high-risk (>50% predicted prevalence), corresponding to the *Ch*ư *Yang Sin* mountain range. This area comprises the eastern half of Lắk district and the southern areas of Krông Bông district where most of the area was not sampled except for three schools located at lower altitude. The predicted risk post-intervention decreased for most of the province, with a slight increase in high-risk areas in the southern part of the province. Predicted MHI *N. americanus* infection risk (categorised as < 1%, 1% to < 2%, 2% to < 5%, and ≥ 5%) is shown in Fig 5. Large portions of the province had a predicted MHI *N. americanus* risk >2%, with considerable reductions in MHI *N. americanus* infection risk across the province post-intervention (Fig 6). The performance of each model was evaluated using K-fold cross-validation. The mean errors of the overall hookworm risk models were 0.135 (SD 0.036) and 0.091 (SD 0.024) pre- and post-intervention, respectively. The mean errors of the MHI *N. americanus* infection risk models were 0.39 (SD 0.011) and 0.017 (SD 0.004) pre- and post-intervention, respectively.

## Discussion

To our knowledge, this is the first study to develop risk prediction maps for hookworm infections in Vietnam. Our results indicate the predicted risk of hookworm infection in most of the province is 10–15%, corresponding to WHO recommendations for continuing school-based PC at an annual frequency [6]. These predicted infection risks are in keeping with prevalence estimates from the CoDe-STH trial [12], but provide additional information in identifying higher-risk areas for hookworm infection and MHI *N. americanus* infection in the northwestern and southern districts. Ongoing transmission in these areas may be due to several factors, including favourable ecological niches borne out by our geospatial modelling, poor school-based PC coverage, or persisting reservoirs of infection that are not targeted by school-based PC programs (e.g., adults and pre-school-aged children). The spatial heterogeneity demonstrated by our risk predictions identifies hotspots, particularly in the Lắk, Krông Bông and M’Đrắk districts, for prioritisation of targeted interventions coupled with enhanced monitoring that would improve resource allocation and program efficiency. In particular, the implementation of community-wide MDA, which has been shown to be a more cost-effective strategy compared to school-based PC to control STH burden in hookworm predominant settings such as Dak Lak, should be considered [14,29]. Continuing PC indefinitely is not considered a sustainable strategy, with the WHO setting a timeline to reduce the number of tablets administered to children by 30% in 2025 and 50% in 2030 [30]. To complement PC strategies, other factors leading to sustained STH prevalence, such as poor WASH practices and infrastructure need to be addressed [7,31], with community-driven WASH interventions shown to reduce open defaecation and *N. americanus* infection risks [32]. The imperative to upscale control efforts is even stronger for central and southern areas of Dak Lak province where the predicted MHI *N. americanus* infection risk is higher than 2%, an important threshold set by WHO to eliminate STH-related disease burden [30]. Of note, there are high-risk areas in the south and southeast corners of the province, where the post-intervention predicted hookworm infection risk is greater than pre-intervention. These areas correspond to distinct geographic features such as a high mountain range where empirical data from the trial were not available to inform our models. Prevalence surveys in these predicted hotspots are warranted to verify our findings given the logistical field work challenges posed by the geography.

Our modelling results reveal that temperature and temperature fluctuations were strong predictors of hookworm infection risk. Contrary to literature about hookworm’s ability to withstand temperatures up to 35–40ºC [33], we found hookworm infection to be less prevalent where the maximum temperature of the warmest month is higher. However, our results concur with laboratory experiments and modelling studies in which hookworms were found to thrive in lower extreme temperatures (annual mean temperature < 27.3ºC and lower land surface temperature) as the larvae are prone to desiccation [18,34,35]. Furthermore, a relationship between soil pH and hookworm prevalence has been suggested in the literature [36]. In our setting, soil pH is near optimal for hookworm eggs to hatch [37], which partly explains the ability of hookworms to thrive in Dak Lak province. This is also the case for vegetation indices, with the optimal NDVI range for hookworms shown to be 0.4 to 0.6 [34,38–40], which is reflected in our analyses where deviation from the optimal range lowered the predicted risk.

Our study comes with limitations. Most importantly, risk predictions derived from our models do not consider population size or density and age distribution. In addition, the at-risk population in areas that were not sampled in the parasitological surveys require further investigation. The unavailability of household-level coordinates, which could have better utilised the high-resolution environmental data, means that spatial variance between households was unable to be captured. Lastly, the short timeframe between pre- and post-intervention surveys (approximately 12 months) provides limited scope to detect an impact from STH control programs.

There are several strengths to our study. The diagnostic tool used for the parasitological surveys was qPCR, which is highly sensitive and able to distinguish between hookworm species, and has been increasingly recognised as the gold standard [41–43]. This analysis also used data obtained in the context of a randomised controlled trial, with a sampling process that included both randomisation of participating schools and randomisation of schools into either community-wide MDA or school-based PC [11]. This minimises the risk of selection bias and increases external validity, which is an important consideration for a predictive task. Aside from discrete areas in the northwestern, south and southeastern corners of the province, sampling locations were relatively evenly disbursed throughout the province, with representation from all districts. As a result, our predictors are likely to have captured a large part of the spatial variance among the study samples.

In conclusion, the current school PC program in Dak Lak should be continued at least at the current annual frequency. However, our risk prediction models have identified several areas at higher risk for hookworm infection and hotspots of MHI *N. americanus* infection in school-aged children, particularly in the southern and eastern parts of the province. Targeted interventions, such as increased school PC frequency or community-wide MDA, complemented by WASH interventions, and coupled with an enhanced monitoring and evaluation program, should be considered for these hotspots.

## Supporting information

S1 Table Pre- and post-intervention prevalence of overall hookworm infections and moderate and-heavy *Necator americanus* infections across study sites. (PDF)

S2 Table Summary statistics of environmental and bioclimatic characteristics across Dak Lak province and at sampled schools (N = 64). (PDF)

S3 Table Univariable analysis of bioclimatic variables for hookworm infection and moderate-and-heavy intensity *Necator americanus* infection. (PDF)

S1 Fig Semivariograms for overall hookworm infection risk and moderate-and-heavy intensity *Necator americanus* infection risk models. (PDF)
