# Supplementary material for: Geospatial risk prediction of hookworm infection and intensity among school-aged children in Dak Lak province, Vietnam
Source: PLoS Negl Trop Dis. 2026 Mar 12;20(3):e0014079. doi: 10.1371/journal.pntd.0014079 (PMC13004524; doi:10.1371/journal.pntd.0014079)
Supplement: S1 Table — (PDF) [file pntd.0014079.s001.pdf]

S1 Table. Pre- and post-intervention prevalence of overall hookworm infections and moderate-and-heavy *Necator americanus* infections across study sites.

			Pre-intervention		Post-intervention	
**Study site**	**Trial arm**	**N**	**Hookworm n (%)**	**MHI *N. americanus* n (%)**	**Hookworm n (%)**	**MHI *N. americanus* n (%)**
1	School PC	123	9 (7.32)	2 (1.63)	6 (4.88)	0 (0.00)
2	School PC	122	35 (28.69)	7 (5.74)	18 (14.75)	3 (2.46)
3	School PC	120	20 (16.67)	7 (5.83)	10 (8.33)	0 (0.00)
4	School PC	119	18 (15.13)	2 (1.68)	16 (13.45)	1 (0.84)
5	School PC	70	20 (28.57)	8 (11.43)	21 (30.00)	4 (5.71)
6	School PC	118	1 (0.85)	0 (0.00)	0 (0.00)	0 (0.00)
7	School PC	119	11 (9.24)	0 (0.00)	1 (0.84)	0 (0.00)
8	School PC	115	12 (10.43)	3 (2.61)	7 (6.09)	0 (0.00)
9	School PC	120	6 (5.00)	0 (0.00)	0 (0.00)	0 (0.00)
10	School PC	118	29 (24.58)	2 (1.69)	16 (13.56)	4 (3.39)
11	School PC	125	3 (2.40)	0 (0.00)	1 (0.80)	0 (0.00)
12	School PC	120	26 (21.67)	2 (1.67)	22 (18.33)	4 (3.33)
13	School PC	119	45 (37.82)	15 (12.61)	37 (31.09)	15 (12.61)
14	School PC	120	35 (29.17)	8 (6.67)	32 (26.67)	4 (3.33)
15	School PC	123	36 (29.27)	4 (3.25)	19 (15.45)	3 (2.44)
16	School PC	120	5 (4.17)	0 (0.00)	6 (5.00)	2 (1.67)
17	School PC	122	7 (5.74)	3 (2.46)	2 (1.64)	0 (0.00)
18	School PC	118	10 (8.47)	2 (1.69)	5 (4.24)	0 (0.00)
19	School PC	120	35 (29.17)	3 (2.50)	16 (13.33)	1 (0.83)
20	School PC	125	2 (1.60)	1 (0.80)	1 (0.80)	0 (0.00)
21	School PC	124	7 (5.65)	2 (1.61)	3 (2.42)	0 (0.00)
22	School PC	118	1 (0.85)	0 (0.00)	1 (0.85)	0 (0.00)
23	School PC	135	76 (56.30)	22 (16.30)	52 (38.52)	8 (5.93)
24	School PC	122	27 (22.13)	8 (6.56)	18 (14.75)	7 (5.74)
25	School PC	127	12 (9.45)	2 (1.57)	4 (3.15)	0 (0.00)
26	School PC	119	19 (15.97)	0 (0.00)	8 (6.72)	0 (0.00)
27	School PC	123	5 (4.07)	0 (0.00)	1 (0.81)	0 (0.00)
28	School PC	119	21 (17.65)	10 (8.40)	11 (9.24)	6 (5.04)
29	School PC	120	53 (44.17)	16 (13.33)	25 (20.83)	3 (2.50)
30	School PC	120	11 (9.17)	2 (1.67)	13 (10.83)	4 (3.33)
31	School PC	124	1 (0.81)	0 (0.00)	2 (1.61)	0 (0.00)
32	School PC	122	41 (33.61)	4 (3.28)	22 (18.03)	2 (1.64)
33	Community MDA	121	23 (19.01)	9 (7.44)	27 (22.31)	1 (0.83)

Study site	Trial arm	N	Pre-intervention		Post-intervention	
			**Hookworm n (%)**	**MHI *N. americanus* n (%)**	**Hookworm n (%)**	**MHI *N. americanus* n (%)**
34	Community MDA	122	17 (13.93)	3 (2.46)	18 (14.75)	4 (3.28)
35	Community MDA	124	12 (9.68)	4 (3.23)	2 (1.61)	0 (0.00)
36	Community MDA	122	17 (13.93)	12 (9.84)	13 (10.66)	0 (0.00)
37	Community MDA	120	4 (3.33)	0 (0.00)	4 (3.33)	0 (0.00)
38	Community MDA	120	8 (6.67)	0 (0.00)	0 (0.00)	0 (0.00)
39	Community MDA	120	1 (0.83)	0 (0.00)	1 (0.83)	0 (0.00)
40	Community MDA	127	70 (55.12)	22 (17.32)	35 (27.56)	1 (0.79)
41	Community MDA	125	10 (8.00)	3 (2.40)	7 (5.60)	1 (0.80)
42	Community MDA	104	6 (5.77)	1 (0.96)	7 (6.73)	2 (1.92)
43	Community MDA	123	9 (7.32)	1 (0.81)	4 (3.25)	0 (0.00)
44	Community MDA	123	18 (14.63)	5 (4.07)	9 (7.32)	2 (1.63)
45	Community MDA	115	15 (13.04)	6 (5.22)	8 (6.96)	1 (0.87)
46	Community MDA	120	2 (1.67)	0 (0.00)	1 (0.83)	0 (0.00)
47	Community MDA	119	1 (0.84)	0 (0.00)	2 (1.68)	0 (0.00)
48	Community MDA	124	9 (7.26)	1 (0.81)	0 (0.00)	0 (0.00)
49	Community MDA	118	34 (28.81)	4 (3.39)	19 (16.10)	0 (0.00)
50	Community MDA	121	16 (13.22)	4 (3.31)	8 (6.61)	0 (0.00)
51	Community MDA	124	7 (5.65)	2 (1.61)	6 (4.84)	0 (0.00)
52	Community MDA	122	1 (0.82)	0 (0.00)	0 (0.00)	0 (0.00)
53	Community MDA	121	3 (2.48)	0 (0.00)	2 (1.65)	0 (0.00)
54	Community MDA	120	28 (23.33)	5 (4.17)	21 (17.50)	1 (0.83)
55	Community MDA	120	4 (3.33)	0 (0.00)	5 (4.17)	1 (0.83)
56	Community MDA	127	18 (14.17)	5 (3.94)	16 (12.60)	3 (2.36)
57	Community MDA	130	6 (4.62)	0 (0.00)	3 (2.31)	0 (0.00)
58	Community MDA	119	1 (0.84)	0 (0.00)	2 (1.68)	0 (0.00)
59	Community MDA	118	0 (0.00)	0 (0.00)	1 (0.85)	0 (0.00)
60	Community MDA	121	9 (7.44)	0 (0.00)	6 (4.96)	0 (0.00)
61	Community MDA	120	4 (3.33)	3 (2.50)	3 (2.50)	0 (0.00)
62	Community MDA	115	40 (34.78)	10 (8.70)	19 (16.52)	1 (0.87)
63	Community MDA	120	8 (6.67)	1 (0.83)	5 (4.17)	1 (0.83)
64	Community MDA	120	49 (40.83)	14 (11.67)	9 (7.50)	1 (0.83)

PC = preventive chemotherapy. MDA = mass drug administration. MHI = moderate-to-heavy intensity.
