# Supplementary material for: Geospatial risk prediction of hookworm infection and intensity among school-aged children in Dak Lak province, Vietnam
Source: PLoS Negl Trop Dis. 2026 Mar 12;20(3):e0014079. doi: 10.1371/journal.pntd.0014079 (PMC13004524; doi:10.1371/journal.pntd.0014079)
Supplement: S2 Table — (PDF) [file pntd.0014079.s002.pdf]

**S2 Table.** Summary statistics of environmental and bioclimatic characteristics across Dak Lak province and at sampled schools (N = 64).

| Characteristic | Province estimates | | Study location estimates | |
|---|---|---|---|---|
| | Median (IQR) | Range (min, max) | Median (IQR) | Range (min, max) |
| **Temperature (°C) and elevation (m)** | | | | |
| Elevation (m) | 453 (263, 640) | 16, 2279 | 465 (426, 539) | 172, 823 |
| Annual Mean Temperature | 24 (22.7, 25.3) | 14.2, 26.8 | 23.9 (23.4, 24.2) | 22.1, 26.2 |
| Mean Diurnal Range | 7.8 (7.4, 8.1) | 6.3, 9.6 | 8.1 (7.9, 8.2) | 7.0, 8.5 |
| Isothermality | 57.6 (56.7, 58.9) | 52.1, 63.8 | 57.8 (57.3, 58.1) | 55.7, 62.2 |
| Temperature Seasonality | 163.5 (155.3, 177.5) | 112.1, 230.9 | 163.3 (157.8, 175.0) | 148.3, 196.8 |
| Max Temperature of Warmest Month | 30.7 (29.2, 31.8) | 20.4, 33.1 | 30.9 (30.1, 31.2) | 29.1, 32.9 |
| Min Temperature of Coldest Month | 17.1 (15.6, 18.6) | 7.0, 20.4 | 17 (16.0, 17.0) | 15.0, 19.0 |
| Temperature Annual Range | 13.4 (13.0, 14.0) | 11.6, 15.2 | 14 (14.0, 14.0) | 12.0, 15.0 |
| Mean Temperature of Wettest Quarter | 24.3 (23.1, 25.6) | 14.7, 27.3 | 24.1 (23.7, 24.7) | 22.9, 27.0 |
| Mean Temperature of Driest Quarter | 22.6 (21.2, 23.8) | 12.6, 25.6 | 22.7 (22.1, 23) | 20.9, 25.3 |
| Mean Temperature of Warmest Quarter | 25.8 (24.4, 27.1) | 15.4, 29.0 | 25.7 (25.2, 26) | 23.8, 27.9 |
| Mean Temperature of Coldest Quarter | 21.6 (20.4, 22.9) | 12.6, 24.4 | 21.6 (21, 21.9) | 19.8, 24.1 |
| **Precipitation** | | | | |
| Annual Precipitation (cm) | 164.3 (135.6, 179.5) | 112.1, 227.7 | 157.9 (139.7, 169.8) | 127.4, 186.0 |
| Precipitation of Wettest Month (cm) | 28.4 (25.4, 31.3) | 22.0, 49.6 | 26.7 (24.1, 29.8) | 22.3, 34.3 |
| Precipitation of Driest Month (cm) | 0.5 (0.3, 1.1) | 0.1, 2.4 | 0.5 (0.3, 0.8) | 0.1, 1.5 |
| Precipitation Seasonality | 76.7 (70.7, 83.1) | 59.2, 98.7 | 74.7 (69.7, 80) | 66.0, 88.6 |
| Precipitation of Wettest Quarter (cm) | 76.4 (67.2, 90.0) | 58.8, 136.0 | 72.4 (63.9, 85.2) | 60.1, 101.7 |
| Precipitation of Driest Quarter (cm) | 3.5 (2.6, 5.1) | 1.3, 10.1 | 3.0 (2.6, 3.3) | 2.1, 6.0 |
| Precipitation of Warmest Quarter (cm) | 46.9 (35.3, 51.1) | 18.7, 68.0 | 45.4 (38.7, 48.5) | 29.5, 50.7 |
| Precipitation of Coldest Quarter (cm) | 9.1 (5.3, 11.7) | 1.6, 27.7 | 6.4 (4.1, 10.8) | 2.3, 13.9 |
| **Soil characteristics** | | | | |
| Soil pH | 5.8 (5.6, 5.9) | 5.1, 6.3 | 5.8 (5.7, 5.9) | 5.5, 6.1 |
| Soil sand (g/kg) | 35 (34, 37) | 29, 43 | 37 (35, 39) | 31, 41 |
| Soil silt (g/kg) | 31 (30, 32) | 21, 40 | 31 (30, 32) | 27, 36 |
| Soil organic carbon (dg/kg) | 31.6 (27, 40.4) | 17.8, 90.6 | 28 (25.5, 30.2) | 21.7, 39.2 |
| **Vegetation** | | | | |
| Normalised difference vegetation index | 0.60 (0.49, 0.69) | –0.05, 0.93 | 0.59 (0.52, 0.65) | 0.35, 0.72 |
| Enhanced vegetation index | 0.37 (0.31, 0.45) | –0.02, 0.68 | 0.35 (0.31, 0.40) | 0.21, 0.51 |

IQR = interquartile range.
