# Supplementary material for: Geospatial risk prediction of hookworm infection and intensity among school-aged children in Dak Lak province, Vietnam
Source: PLoS Negl Trop Dis. 2026 Mar 12;20(3):e0014079. doi: 10.1371/journal.pntd.0014079 (PMC13004524; doi:10.1371/journal.pntd.0014079)
Supplement: S3 Table — (PDF) [file pntd.0014079.s003.pdf]

S3 Table. Univariable analysis of bioclimatic variables for hookworm infection and moderate-and-heavy intensity *Necator americanus* infection.

Variable	Hookworm infection P-value	Hookworm infection OR (95%CI)	Hookworm infection AIC	*Necator americanus* MHI infection P-value	*Necator americanus* MHI infection OR (95%CI)	*Necator americanus* MHI infection AIC
Elevation (metres) †	0.95	1.00 (1.00, 1.00)	1361	0.13	1.00 (1.00, 1.00)	526
Annual Mean Temp. (°C)	0.002	0.89 (0.82, 0.96)	1351	0.002	0.79 (0.67, 0.91)	518
Mean Diurnal Range (°C)	< 0.001	0.39 (0.32, 0.47)	1275	< 0.001	0.50 (0.34, 0.73)	516
Isothermality	< 0.001	0.89 (0.83, 0.95)	1348	0.11	0.90 (0.79, 1.02)	526
Temp. Seasonality (°C)	< 0.001	1.01 (1.01, 1.02)	1347	0.016	1.01 (1.00, 1.03)	523
Max Temp. of Warmest Month (°C)	< 0.001	0.80 (0.74, 0.86)	1322	< 0.001	0.71 (0.62, 0.82)	507
Min Temp. of Coldest Month (°C)	0.12	0.95 (0.89, 1.01)	1359	0.006	0.84 (0.74, 0.95)	521
Temp. Annual Range (°C) †	< 0.001	0.57 (0.50, 0.65)	1289	0.002	0.67 (0.53, 0.86)	519
Mean Temp. of Wettest Quarter (°C)	< 0.001	0.85 (0.80, 0.91)	1340	< 0.001	0.77 (0.67, 0.89)	515
Mean Temp. of Driest Quarter (°C)	< 0.001	0.86 (0.80, 0.92)	1342	< 0.001	0.76 (0.66, 0.88)	514
Mean Temp. of Warmest Quarter (°C)	0.003	0.89 (0.83, 0.96)	1352	0.002	0.79 (0.68, 0.92)	519
Mean Temp. of Coldest Quarter (°C)	< 0.001	0.87 (0.81, 0.94)	1347	< 0.001	0.77 (0.66, 0.89)	516
Annual Precipitation (mm)	< 0.001	1.00 (1.00, 1.00)	1338	0.040	1.00 (1.00, 1.00)	524
Precipitation of Wettest Month (mm) ‡	0.082	1.00 (1.00, 1.00)	1358	0.54	1.00 (1.00, 1.00)	528
Precipitation of Driest Month (mm)	< 0.001	1.04 (1.02, 1.06)	1342	0.026	1.04 (1.00, 1.08)	524
Precipitation Seasonality (mm) ‡	0.039	0.99 (0.98, 1.00)	1357	0.20	0.99 (0.97, 1.01)	527
Precipitation of Wettest Quarter (mm) ‡	0.016	1.00 (1.00, 1.00)	1355	0.32	1.00 (1.00, 1.00)	528
Precipitation of Driest Quarter (mm)	< 0.001	1.02 (1.02, 1.03)	1326	< 0.001	1.02 (1.01, 1.04)	517
Precipitation of Warmest Quarter (mm)	< 0.001	1.00 (1.00, 1.00)	1323	0.002	1.00 (1.00, 1.00)	519
Precipitation of Coldest Quarter (mm)	< 0.001	1.01 (1.00, 1.01)	1321	0.001	1.01 (1.00, 1.01)	518
Soil pH ‡	0.012	1.75 (1.13, 2.71)	1355	0.36	1.49 (0.64, 3.53)	528
Soil sand content (%) †‡	0.60	0.99 (0.97, 1.02)	1361	0.71	0.99 (0.94, 1.04)	528
Soil silt (%) †‡	0.24	1.02 (0.99, 1.06)	1360	0.85	0.99 (0.93, 1.06)	528
Soil organic carbon (%)	0.034	1.02 (1.00, 1.04)	1357	0.043	1.04 (1.00, 1.07)	525
NDVI [–1, 1] †‡	0.69	0.88 (0.46, 1.69)	1361	0.65	1.35 (0.37, 5.01)	528
EVI [–1, 1] ‡	0.002	0.24 (0.09, 0.60)	1352	0.76	0.75 (0.12, 4.80)	528

AIC = Akaike information criterion. CI = confidence interval. MHI = moderate-and-heavy intensity. OR = odds ratio.

†Variable included in multivariable hookworm model.

‡ Variable included in MHI *N. americanus* model
