# Supplementary material for: Geospatial risk prediction of hookworm infection and intensity among school-aged children in Dak Lak province, Vietnam
Source: PLoS Negl Trop Dis. 2026 Mar 12;20(3):e0014079. doi: 10.1371/journal.pntd.0014079 (PMC13004524; doi:10.1371/journal.pntd.0014079)
Supplement: S1 Fig — (PDF) [file pntd.0014079.s004.pdf]

S1 Fig. Semivariograms for overall hookworm infection risk and moderate-and-heavy intensity *Necator americanus* infection risk models.

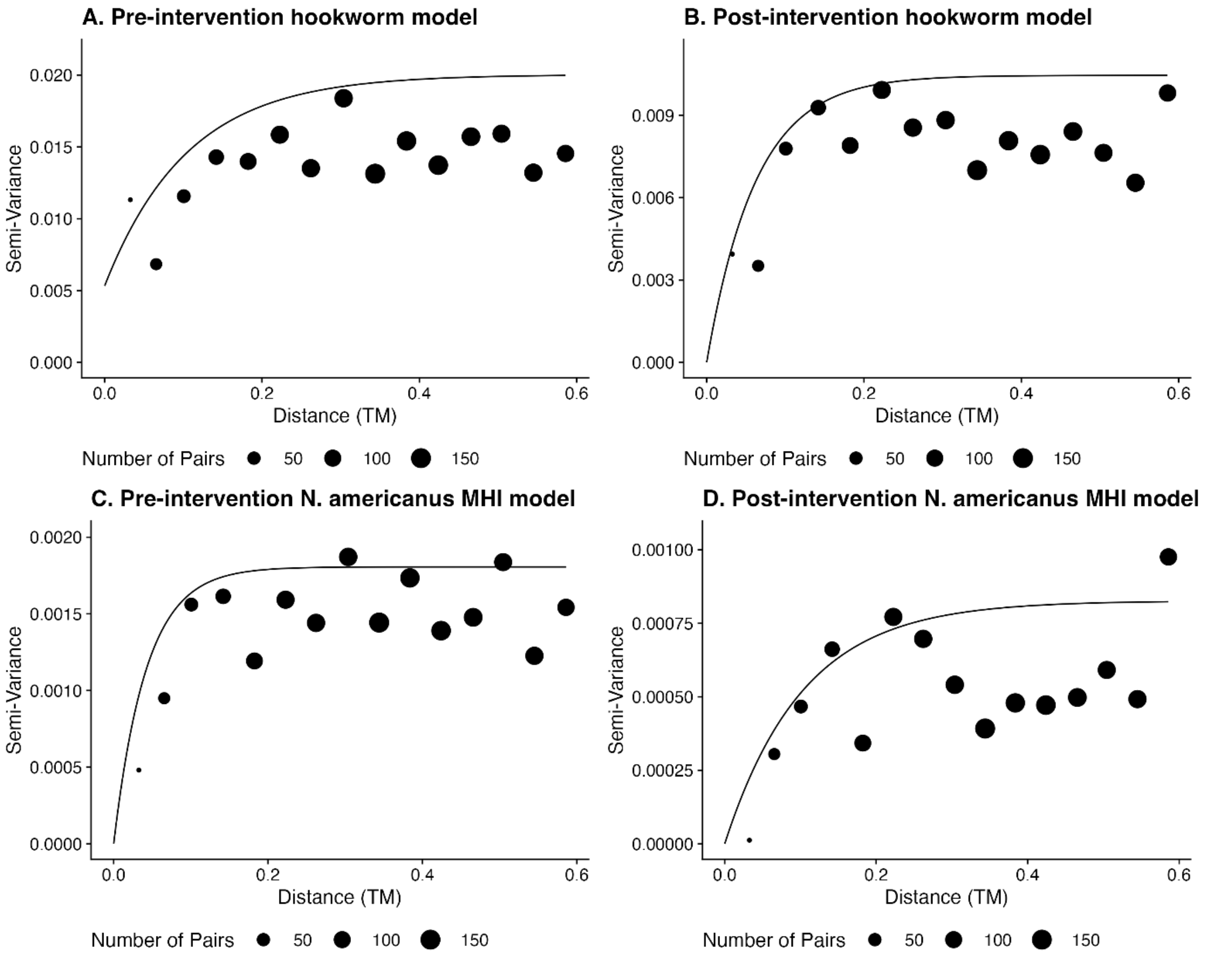

MHI = moderate-and-heavy intensity.
